# Effects of letrozole on serum estradiol and estrone in postmenopausal breast cancer patients and tolerability of treatment: a prospective trial using a highly sensitive LC–MS/MS (liquid chromatography–tandem mass spectrometry) method for estrogen measurement

**DOI:** 10.1007/s10549-023-07054-3

**Published:** 2023-07-25

**Authors:** Mária Faltinová, Leena Vehmanen, Heli Lyytinen, Mikko Haanpää, Esa Hämäläinen, Aila Tiitinen, Carl Blomqvist, Johanna Mattson

**Affiliations:** 1 Comprehensive Cancer Center, Helsinki University Hospital, PO Box 180, 00290 Helsinki, Finland; 2 HUSLAB, Helsinki University Hospital, Helsinki, Finland; 3 University of Helsinki, Helsinki, Finland; 4 Department of Clinical Chemistry, University of Eastern Finland, Kuopio, Finland

**Keywords:** Aromatase inhibitor, Letrozole, Breast cancer, Estradiol, Liquid chromatography tandem mass spectrometry, Quality of life

## Abstract

**Purpose:**

To analyze serum estradiol (E2) and estrone (E1) during letrozole treatment and their association to Quality of Life (QoL) and side-effects.

**Methods:**

Postmenopausal breast cancer patients starting adjuvant letrozole were eligible. Serum samples were taken at baseline, three, and 12 months. E2 and FSH were measured with routine chemiluminescent immunoassays. E2 and E1 were analyzed after trial completion with a highly sensitive liquid chromatography-tandem mass spectrometry method (LC–MS/MS) with lower limits of quantification (LLOQ) of 5 pmol/L. QoL was measured at baseline and at 12 months with the EORTC QLQ-C30 and QLQ-BR23 and the Women’s Health questionnaires, and menopause-related symptoms with the modified Kupperman Index.

**Results:**

Of 100 screened patients 90 completed the trial. Baseline mean LC–MS/MS E2 and E1 were 12 pmol/L (range < 5–57) and 66 pmol/L (< 5–226), respectively. E2 levels measured by immunoassay and LC–MS/MS showed no correlation. E2 and E1 were completely suppressed by letrozole except for one occasion (E1 11 pmol/L at 3 months). Pain, side effects of systemic therapy, vasomotor symptoms, joint and muscle aches, and vaginal dryness increased during letrozole treatment. A high baseline E2 was significantly associated with increased aching joints and muscles, but not with the other side effects.

**Conclusions:**

Letrozole supresses E2 and E1 completely below the LLOQ of the LC–MS/MS in postmenopausal women. High pre-treatment E2 levels were associated with more joint and muscle pain during letrozole. Automated immunoassays are unsuitable for E2 monitoring during letrozole therapy due to poor sensitivity.

**Supplementary Information:**

The online version contains supplementary material available at 10.1007/s10549-023-07054-3.

## Introduction

Hormone receptor-positive [i.e., estrogen (ER) and/or progesterone (PR) receptor-positive] cancers account for 75 percent of all breast cancer cases. Adjuvant endocrine therapy blocks ER function or lowers estrogen levels, reduces the risk of recurrence, and improves survival among women with hormone receptor-positive early breast cancer.

Aromatase inhibitors (AIs) suppress plasma estrogen levels by inhibiting peripheral conversion of androgens to estrogens and reduce breast cancer recurrence and mortality. AIs are the preferred adjuvant therapy for postmenopausal women with hormone receptor-positive breast cancer [1].

Adjuvant AI treatment is often recommended for five to ten years according to the risk of cancer relapse [2]. However, adverse effects of the treatment may negatively impact treatment compliance and Quality of Life (QoL) of the patients [3]. Non-adherence to AI treatment is a well-documented problem, which may increase the risk of relapse and have a negative effect on survival [4, 5].

In postmenopausal women, levels of circulating E2 are typically within the range of 10 to 60 pmol/L [6]. During treatment with AIs, the E2 levels in patients fall to less than 1–3 pmol/L [7, 8]. The sensitivity of the commercial immunoassays (IAs) of estrogens that are used in routine laboratories cannot usually compete with the sensitivity of modern LC–MS/MS methods as they do not usually reach the level of the low picomolar E2 range in postmenopausal women. Thus, they may not be capable of monitoring the changes of estradiol at low postmenopausal concentrations needed for clinical monitoring of AI treatment. It may also be impossible to use them for monitoring for instance the effect of intravaginal estradiol use on circulating E2 levels during adjuvant therapy with Ais.

The primary aim of the present study was to analyze the effects of letrozole treatment on serum estrogens, especially E2, in postmenopausal breast cancer patients, using a highly sensitive and specific LC–MS/MS method. The aim was also to assess our routine chemiluminescent E2 method for monitoring of serum estradiol in post-menopausal patients and compare it to our sensitive LC–MS/MS. Secondary objectives were to analyze the effects of letrozole on serum E1 and to investigate the impact of baseline demographics, E2, E1, and FSH on QoL and treatment tolerability during adjuvant letrozole treatment.

## Patients and methods

This study was carried out at the Helsinki University Central Hospital Comprehensive Cancer Center from October 2015 to January 2017. Eligible patients were postmenopausal women with hormone receptor-positive early-stage breast cancer for whom adjuvant letrozole treatment was planned. Prior adjuvant chemotherapy was not allowed. The patients were classified as postmenopausal if they were either ≥ 60 years of age or had a history of amenorrhea for at least 12 months with serum E2 and FSH levels within the postmenopausal range. Adjuvant treatment with letrozole 2.5 mg daily was started in all patients.

The levels of E2 and FSH were analyzed by a solid-phase, enzyme-labeled chemiluminescent immunoassay in an Immulite 2000 Xpi analyzer (Siemens Healthineers, Tarrytown, NY USA) at three and 12 months during letrozole treatment. After the completion of the trial the levels of serum E2 and E1 were studied by a highly sensitive LC–MS/MS using duplicate serum samples taken at the same time as routine monitoring samples during baseline and at three and 12 months of letrozole treatment and stored in a deep freezer (− 80 C) until analysis.

The patients filled structured QoL and side-effect questionnaires at the start of letrozole treatment and after 12 months. The scores at the start of treatment and after 12 months were calculated.

The study was approved by the local ethics committee at the Helsinki University Hospital. Informed consent was obtained from each participant.

### Quantification of estrogens by LC–MS/MS

Serum E1 and E2 concentrations were analyzed by a LC–MS/MS: Agilent 1200 high-performance liquid chromatography (Agilent Technologies Inc., Santa Clara, CA, USA) coupled with an AB Sciex Triple Quad 5500 mass spectrometer controlled by Analyst Software 1.6.2 (AB Sciex, Concord, ON, Canada). Slight modifications of our previous method were included [9, 10] to optimize the sensitivity and specificity for both serum estrogens, E2 and E1.

Assay calibrators and blank solutions of 0.0–1000 pmol/L E1 (Vetranal, Sigma-Aldrich, St. Louis, MO, USA) and 0.0–1275 pmol/L E2 (Sigma-Aldrich) were prepared in water:methanol (1:1, v/v). To 300 µL of calibrator or 300 µL of serum 30 µL of internal standard (IS) containing 3 nmol/L ^13^C_3_-E1, 3 nmol/L ^13^C_3_-E2 (IsoSciences, Ambler, PA, USA) in water:methanol (19:1, v/v) was added. A serum sample or an assay calibrator with IS was extracted by 1 mL of diethyl ether (DEE). The DEE phase was transferred into a vial and evaporated to dryness after centrifugation. Three hundred µL of 0.1% ammonia water (Sigma-Aldrich) and 1 mL of DEE were added to the residue followed by a second extraction and evaporation of the DEE phase after centrifugation. The serum sample or the assay calibrator residue was dissolved in 125 µL of water:methanol (1:1, v/v).

One hundred µl of the calibrator or the sample extract was measured in one LC–MS/MS run. Chromatographic separation was performed on a tandem column where a Discovery HS F5-3 column (2.1 × 100 mm, 3 µm; Supelco, Bellefonte, PA, USA) was coupled with a SunFire C18 column (2.1 × 50 mm, 3.5 µm; Waters, Milford, MA, USA). The mobile phase was a linear gradient consisting of 40 µmol/L ammonium fluoride in water (A) and methanol (B) at a flow rate of 300 µL/min. The gradient was 0 min 50% B, 4.5–10 min 100% B, and 10.5–19 min 50% B.

E1, E2, and corresponding ^13^C_3_-labelled internal standards were detected for duplicate quantitation by multiple reaction monitoring in the negative ion [M−H]^−^ mode using the following parent ions and selected transitions: E1 [M−H]^−^ *m*/*z* 269.1 to *m*/*z* 269.1 and *m*/*z* 145.0; E2 [M−H]^−^ *m*/*z* 271.2 to *m*/*z* 271.2 and *m*/*z* 183.1; ^13^C_3_-E1 [M−H]^−^ *m*/*z* 272.1 to *m*/*z* 272.1 and *m*/*z* 148.0; ^13^C_3_-E2 [M−H]^−^ *m*/*z* 274.2 to *m*/*z* 274.2 and *m*/*z* 186.1.

Using Analyst Software 1.6.2 data processing tools for assay calibrators and sample quantifications we estimated the lower limits of quantification (LLOQ) to 5 pmol/L for E1 and E2 with signal to noise ratios *S*/*N* to 10 or higher.

### Assessments of health-related Quality of Life (QoL) and menopausal symptoms

QoL data were obtained using the European Organisation for Research and Treatment of Cancer Quality of Life Questionnaire Version 3.0 (EORTC QLQ-C30) and the EORTC Breast Cancer Module questionnaire (QLQ-BR23).The EORTC QLQ-C30 is composed of a global health status/QoL scale, five scales measuring physical, role, emotional, cognitive, and social functioning, three symptom scales (fatigue, nausea/vomiting, and pain), and six single items (dyspnea, insomnia, appetite loss, constipation, diarrhea, and financial difficulties) [11]. The EORTC QLQ-BR23 consists of 23 items that assess breast cancer symptoms, side effects of the treatment, body image, sexual functioning, sexual enjoyment, and future perspective [12]. Based on standard EORTC scoring procedures, all scales were linearly converted to a scale from 0 to 100. For scales evaluating global health and functioning, higher scores represent higher levels of functioning and health status. For the evaluation of symptoms, higher scores correspond to more symptoms.

The Women’s Health Questionnaire (WHQ) was used to measure health-related quality of life, psychological well-being, and emotional well-being. This questionnaire which has been validated in Finnish language is a commonly used instrument for measuring climacteric-related symptoms [13]. It is a self-administered questionnaire composed of 36 items capturing nine domains of women’s health: vasomotor symptoms (hot flushes and night sweats), somatic symptoms (headaches, tiredness, dizzy spells, pain in limbs or back, nausea, pins and needles in hands or feet, and frequent need to pass urine), anxiety and fears (four items), depression (seven items), sleep problems (three items), sexual behaviour (three items), memory and concentration (three items), menstrual cycle-related symptoms (four items), and attractiveness (three items). Each item is answered on a four-point scale (1–4) and then reduced to a binary scale (1 and 2 = 0, 3 and 4 = 1) for scoring. A mean score (between 0 and 1) was calculated for each domain (of the corresponding items), and thus the higher the score, the better the Quality of Life.

Menopausal symptoms during the past two weeks were assessed with a modified Kupperman Index [14, 15]. It comprises 19 items answered on a four-point scale regarding the frequency of night sweats, hot flushes, numbness, insomnia, irritability, a feeling of exhaustion, depressive mood, dizziness, weakness, aching joints or muscles, headache, palpitation, vaginal dryness, oedema, shortness of breath, dryness of mouth, a feeling of a lump in the throat, nausea, and trembling. The incidence of the symptoms was scored as follows: 1, seldom/not at all; 2, once a month; 3, once a week; 4, almost every day. Thus, the higher the score, the more frequent the symptom.

### Statistical methods

The data was analysed using SPSS statistic Version 28. Baseline demographics and characteristics of the patients were summarized using median and range, or mean and standard deviation. The association between baseline hormone levels, and between hormone levels and demographic characteristics (age, BMI, weight, waist circumference) was tested with the Pearson correlation coefficient. The association between treatment discontinuation and baseline hormone levels was tested with the unpaired t-test. A Bonferroni correction for multiple testing was used when appropriate.

We tested the effect of the magnitude of change of E2 levels during letrozole treatment in two steps. Firstly, we determined the significance of changes in QoL and symptom scores from baseline to 12 months by a paired t-test. Due to the large number of testable variables a Bonferroni correction was applied, and the level of significance was set as < 0.001. Only scales with significant change during letrozole treatment were tested for association with E2 changes during treatment. The analysis was done by linear regression on score change during treatment by hormone level. In addition to significantly changed single items we also tested the impact of E2 on change in global QoL assessed by the EORTC QLQ-C30 questionnaires.

## Results

**Fig. 1:**
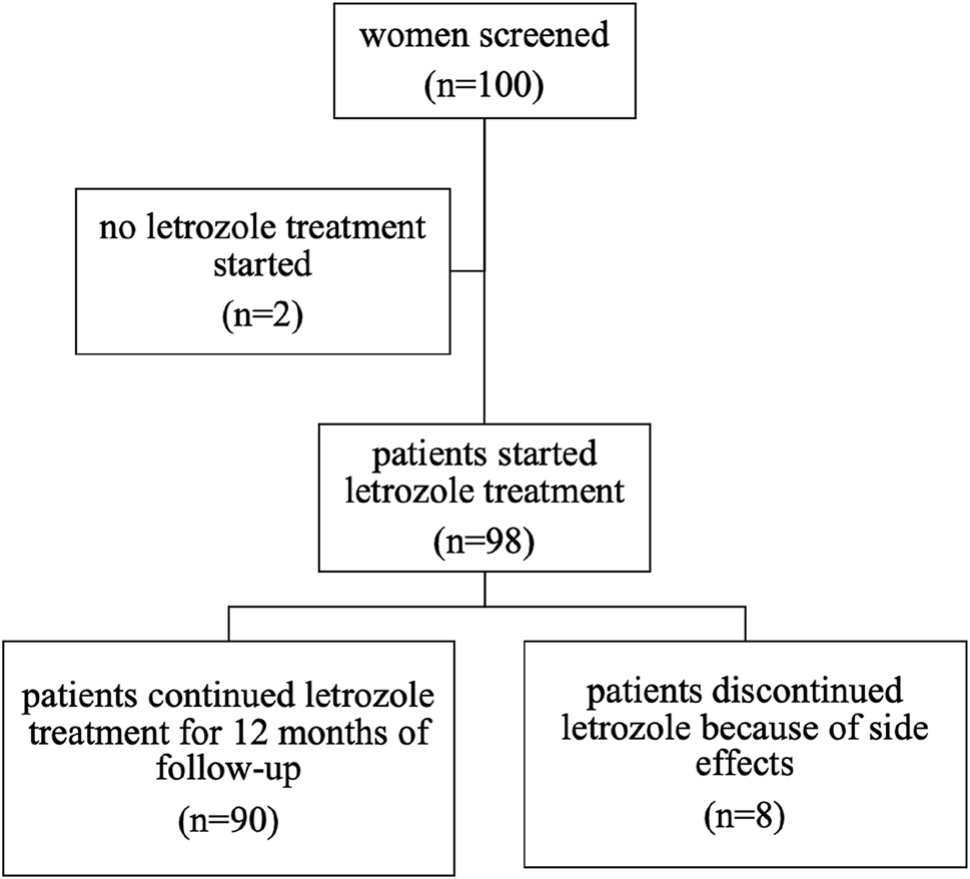
Flow chart of the 100 screened patients

One hundred postmenopausal breast cancer patients with a median age of 66 years (range 54–82 years) were screened for the study. Two patients were excluded since they never started letrozole. After screening, 98 patients started letrozole treatment. Ninety patients (92%) continued letrozole treatment during 12 months of follow-up. Eight patients (8%) discontinued letrozole because of side effects. A flow-chart of the recruitment of the study population is shown in Fig. 1.

Most patients (85%) had T1-tumors, and 85% were node negative. All tumors were ER-positive, and HER2-negative. The baseline characteristics of 90 patients continuing and 8 patients discontinuing letrozole treatment are shown in Table 1. There was no significant association between baseline hormone levels and letrozole discontinuation. However, a high baseline weight and BMI were associated with discontinuation of letrozole therapy. Correlations between baseline hormone levels and demographic factors are shown in Table 2. The baseline serum E2 level analyzed by LC–MS/MS was below 5 pmol/L (LLOQ of our method for E2) in 16 patients. There was no significant association between serum E2 levels measured by immunoassay and LC–MS/MS, (r = 0.17). There was a strong positive correlation between E2 and E1 levels, measured by LC–MS/MS (r = 0.85), a moderate negative correlation between E2 by LC–MS/MS and FSH levels (r = − 0.32), and a weak negative correlation between E1 by LC–MS/MS and FSH levels (r = − 0.27).

**Table 1:** Baseline characteristics of patients continuing and discontinuing letrozole treatment during follow-up

Variable^a^	Patients starting letrozole (n = 98)		
	Patients continuing letrozole (n = 90)	Patients discontinuing letrozole for toxicity (n = 8)	P value
Age at study entry (years)	65 ± 7	68 ± 8	0.23
Age at menarche (years)	13 ± 1	14 ± 2	0.16
Age at menopause (years)	50 ± 5	49 ± 4	0.74
Baseline weight (kg)	69 ± 12	83 ± 16	0.003*
BMI (kg/m^2^)	25 ± 4	30 ± 6	0.004*
Waist circumference (cm)	88 ± 12	101 ± 19	0.008
Baseline FSH (IU/L)	67 ± 23	61 ± 18	0.46
Baseline E2 by IA (pmol/L)	120 ± 33	114 ± 31	0.58
Baseline E2 by LC–MS/MS (pmol/L)	12 ± 10	16 ± 13	0.34
Baseline E1 by LC–MS/MS (pmol/L)	65 ± 40	81 ± 59	0.29
Tumor type			
Ductal	51 (57%)	7 (88%)	
Lobular	19 (21%)	1 (12%)	
Others	20 (22%)	0	
Histological grade			
1	28 (31%)	6 (75%)	
2	51 (57%)	1 (12.5%)	
3	11 (12%)	1 (12.5%)	
Tumor size			
T1	76 (84%)	7 (88%)	
T2	14 (16%)	1 (12%)	
Lymph node status			
N0	76 (84%)	7 (88%)	
N1	14 (16%)	1(12%)	
ER status			
Positive	90 (100%)	8 (100%)	
PgR status			
Positive	70 (78%)	7 (88%)	
Negative	20 (22%)	1 (12%)	
Her-2 status			
Negative	90 (100%)	8 (100%)	
M status			
M0	90 (100%)	8 (100%)	
Ki-67 (%)	14 ± 14	11 ± 13	

*Bonferroni-corrected significance level 0.005

^a^Mean and SD or number and (%)

**Table 2:** Correlations between baseline hormone levels and demographic factors

Correlation	E2 by LC–MS/MS	E1 by LC–MS/MS	FSH	E2 by IA
Age				
Correlation coefficient r	− 0.06	− 0.02	0.01	− 0.07
P value	0.57	0.87	0.9	0.51
Weight				
Correlation coefficient r	0.25	0.16	− 0.34	0.25
P value	0.01	0.11	< 0.001*	0.01
BMI				
Correlation coefficient r	0.22	0.07	− 0.22	0.27
P value	0.03	0.47	0.001*	0.007
Waist circumference				
Correlation coefficient r	0.22	0.13	− 0.33	0.19
P value	0.03	0.21	< 0.001*	0.06
E2 by IA				
Correlation coefficient r	0.17	0.15	− 0.05	
P value	0.09	0.15	0.61	
E1 by LC–MS/MS				
Correlation coefficient r	0.85			
P value	< 0.001*			
FSH				
Correlation coefficient r	− 0.32	− 0.27		
P value	0.001*	0.006		

*Bonferroni-corrected significance level ≤ 0.002

**Fig. 2:**
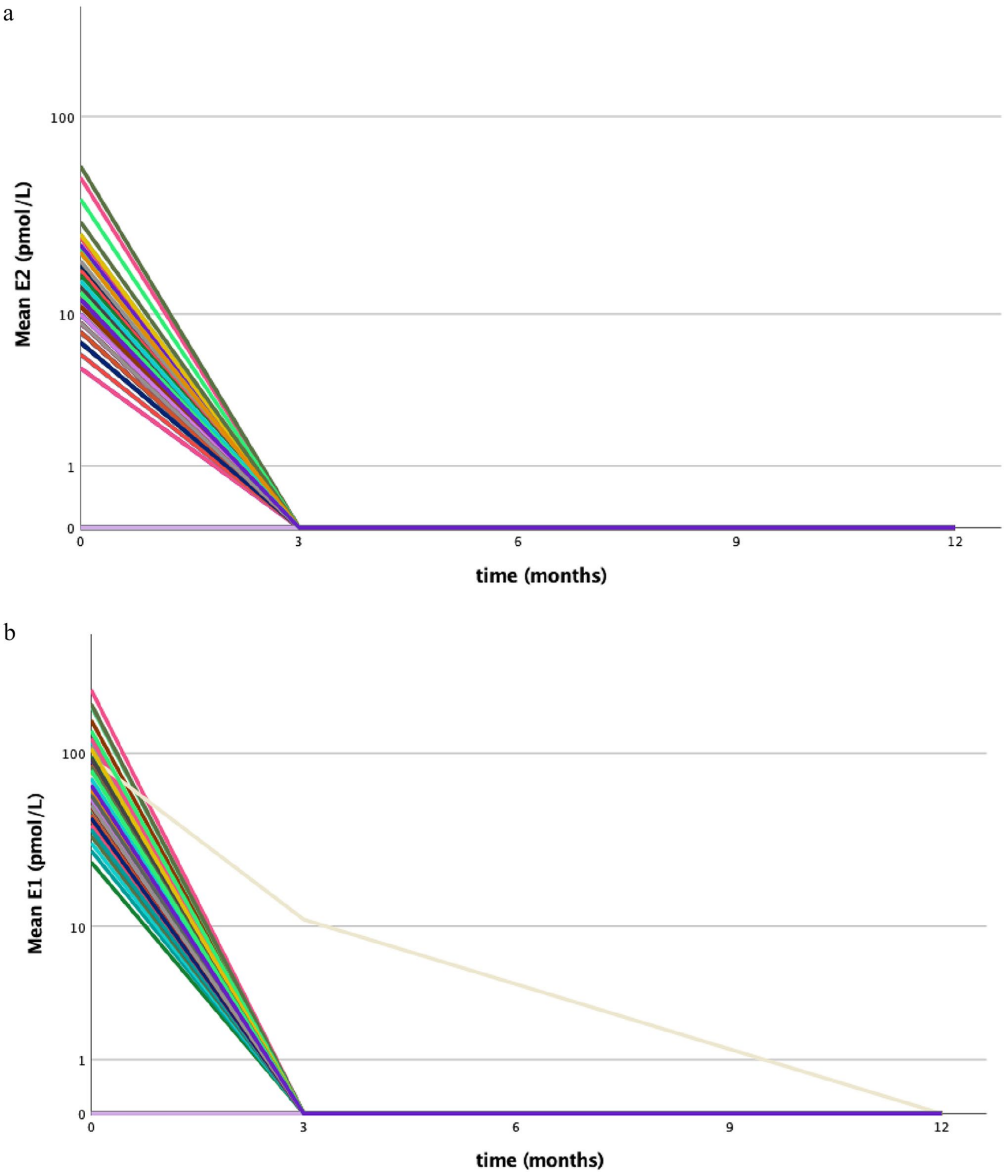
Intra- individual change in **a** E2 levels and **b** E1 levels measured by LC–MS/MS in 90 patients who continued letrozole treatment during follow-up. Lines join log-transformed serum concentrations of E1 and E2 prior to and following 12 months of letrozole treatment. Each line represents a subject

In the following analyses only patients continuing letrozole for 12 months were included (n = 90). In all 90 patients, the levels of serum E2 analyzed by LC–MS/MS were below 5 pmol/L (LLOQ for E1) at three and 12 months of follow-up. Only in one patient serum E1 level remained above 5 pmol/L (11 pmol/L) at three months but decreased below the LLOQ at 12 months. Individual changes in serum E1 and E2 levels in patients are shown in Fig. 2.

QoL and menopausal symptom measures were assessed at baseline and at 12 months after the start of letrozole. Mean QoL scores measured by EORTC QLQ-C30 and EORTC QLQ BR-23 questionnaires at baseline and during the first year of letrozole treatment are shown in Tables 3 and 4. The global EORTC QLQ-C30 health status and all functional scales scores remained stable. Pain (p < 0.001) and the scale *side effects of the therapy* (p < 0.001) increased significantly. In the analysis of the WHQ questionnaire only vasomotor symptoms increased significantly (Table 5). According to the modified Kupperman index, problems with joint or muscle pain (p < 0.001) and vaginal dryness (p < 0.001) increased significantly during the first year of letrozole treatment (Table 6). Moderate or severe joint aches were reported at baseline by 48% and after 12 months of letrozole by 74% of the patients. Similarly, moderate or severe vaginal dryness was reported by 16% at baseline and by 37% after 12 months of letrozole.

**Table 3:** EORTC QLQ-C30 scores at baseline and 12 months after letrozole treatment

Measure	Baseline (n) Mean ± SD	12-months (n) Mean ± SD	P value
Global health scale			
Global health status/QoL	(82) 73 ± 17	(82) 71 ± 20	0.1
Functioning scales			
Physical functioning	(84) 84 ± 16	(84) 82 ± 18	0.07
Role functioning	(83) 87 ± 19	(83) 88 ± 19	0.67
Emotional functioning	(83) 85 ± 18	(83) 83 ± 18	0.45
Cognitive functioning	(82) 88 ± 19	(82) 86 ± 20	0.27
Social functioning	(81) 89 ± 18	(81) 91 ± 19	0.1
Symptom scales			
Fatigue	(84) 24 ± 19	(84) 27 ± 20	0.1
Nausea and vomiting	(83) 2 ± 7	(83) 2 ± 7	0.6
Pain	(81) 14 ± 19	(81) 25 ± 25	< 0.001*
Symptom single items			
Dyspnea	(83) 5 ± 13	(83) 9 ± 16	0.02
Insomnia	(84) 29 ± 31	(84) 37 ± 31	0.007
Appetite loss	(84) 4 ± 12	(84) 5 ± 17	0.52
Constipation	(84) 10 ± 23	(84) 13 ± 25	0.18
Diarrhea	(83) 6 ± 15	(83) 6 ± 14	0.67
Financial difficulties	(82) 12 ± 24	(82) 7 ± 19	0.03

Values are expressed as mean ± SD

*Bonferroni-corrected significance level < 0.001

**Table 4:** EORTC QLQ-BR23 scores at baseline and 12 months after letrozole treatment

Measure	Baseline (n) Mean ± SD	12-months Mean ± SD	P value
Functioning scales			
Body image	(82) 84 ± 20	(82) 82 ± 23	0.25
Sexual functioning	(69) 27 ± 26	(69) 25 ± 25	0.48
Sexual enjoyment	(27) 60 ± 29	(27) 53 ± 27	0.14
Future perspective	(82) 63 ± 26	(82) 59 ± 30	0.19
Symptom scales			
Systemic therapy symptoms	(83) 16 ± 13	(83) 21 ± 17	< 0.001*
Breast symptoms	(83) 17 ± 16	(83) 13 ± 12	0.05
Arm symptoms	(83) 12 ± 16	(83) 16 ± 19	0.12
Upset by hair loss	(12) 17 ± 22	(12) 19 ± 33	0.72

Values are expressed as mean ± SD

*Bonferroni-corrected significance level < 0.001

**Table 5:** Scores for health-related quality of life at baseline and 12 months after letrozole treatment, as measured with the Women’s Health Questionnaire (WHQ)

Women’s heath questionnaire factor	Baseline (n) Mean ± SD	12-months (n) Mean ± SD	P value
Vasomotor symptoms	(79) 0.28 ± 0.36	(79) 0.64 ± 0.43	< 0.001*
Somatic symptoms	(83) 0.31 ± 0.27	(83) 0.39 ± 0.26	0.002
Anxiety and fears	(82) 0.12 ± 0.19	(82) 0.15 ± 0.23	0.21
Depression	(77) 0.15 ± 0.19	(77) 0.24 ± 0.33	0.004
Sleep problems	(82) 0.36 ± 0.3	(82) 0.41 ± 0.32	0.18
Sexual behavior	(37) 0.4 ± 0.36	(37) 0.41 ± 0.37	0.83
Memory and concentration	(81) 0.27 ± 0.32	(81) 0.39 ± 0.36	0.002
Menstrual cycle-related symptoms	(75) 0.24 ± 0.3	(75) 0.31 ± 0.34	0.1
Attractiveness	(83) 0.44 ± 0.3	(83) 0.39 ± 0.33	0.18

Values are expressed as mean ± SD

*Bonferroni-corrected significance level < 0.001

**Table 6:** Prevalence of menopause-related symptoms at baseline and 12 months after letrozole treatment, as measured by the modified Kupperman Index

Menopause-related symptoms	Baseline (n) Mean ± SD	12-months (n) Mean ± SD	P value
Night sweats	(83) 0.51 ± 0.5	(83) 0.58 ± 0.5	0.11
Hot flushes	(82) 0.49 ± 0.5	(82) 0.61 ± 0.5	0.003
Numbness	(81) 0.25 ± 0.43	(81) 0.31 ± 0.47	0.23
Insomnia	(81) 0.52 ± 0.5	(81) 0.51 ± 0.5	0.84
Irritability	(80) 0.23 ± 0.42	(80) 0.29 ± 0.46	0.23
Feeling exhausted	(83) 0.45 ± 0.5	(83) 0.48 ± 0.5	0.47
Depressive mood	(83) 0.13 ± 0.34	(83) 0.19 ± 0.4	0.13
Dizziness	(83) 0.12 ± 0.33	(83) 0.12 ± 0.33	1.0
Weakness	(83) 0.14 ± 0.35	(83) 0.11 ± 0.31	0.41
Aching joints or muscles	(82) 0.46 ± 0.5	(82) 0.73 ± 0.45	< 0.001*
Headache	(82) 0.22 ± 0.42	(82) 0.23 ± 0.43	0.81
Palpitation	(83) 0.23 ± 0.42	(83) 0.17 ± 0.38	0.2
Vaginal dryness	(80) 0.16 ± 0.37	(80) 0.36 ± 0.48	< 0.001*
Oedema	(81) 0.2 ± 0.4	(81) 0.22 ± 0.42	0.66
Shortness of breath	(83) 0.04 ± 0.19	(83) 0.1 ± 0.3	0.02
Dryness of mouth	(81) 0.32 ± 0.47	(81) 0.38 ± 0.49	0.28
A feeling of a lump in the throat	(83) 0.12 ± 0.33	(83) 0.12 ± 0.33	1.0
Nausea	(83) 0.07 ± 0.26	(83) 0.08 ± 0.28	0.71
Trembling	(83) 0.07 ± 0.26	(83) 0.08 ± 0.28	0.66

*Bonferroni-corrected significance level < 0.001

Values are expressed as mean ± SD

The effect of E2 measured by LC–MS/MS on the 12-month changes in global QoL, pain, side effects of systemic therapy, vasomotor symptoms, joint and muscle pain, and vaginal dryness during 12 months of letrozole treatment is shown in Table 7. As E2 measured by LC–MS/MS was unmeasurable at 12 months in all patients, the linear regression was done on baseline hormone levels. A high baseline E2 was significantly associated with increased aching joints and muscles but not with the other side effects. Results of an explorative linear regression analysis of QoL and side-effect changes on baseline FSH and E1 are shown in Tables S1. A low pre-treatment FSH-level predicted more joint pain during treatment, but E1 was not predictive for any of the side-effects.

**Table 7:** The effect of baseline E2 as measured by LC–MS/MS on global QoL, pain, side effects of systemic therapy, vasomotor symptoms, joint or muscle pain, and vaginal dryness during 12 months of letrozole

Dependent variables	Independent variables	Standardized coefficient	P-value
QoL (EORT QLQ-C30)^a^	E2	− 0.02	0.89
Pain (EORT QLQ-C30)^b^	E2	− 0.05	0.66
Side effects of systemic therapy (EORT QLQ-BR23)^b^	E2	− 0.02	0.87
Vasomotor symptoms (WHQ)^c^	E2	− 0.08	0.48
Aching joints and muscles (KI)^b^	E2	0.30	0.006*
Vaginal dryness (KI)^b^	E2	− 0.15	0.17

*A Bonferroni-corrected-p-value threshold of 0.01 was used in the regression analyses

^a^A positive coefficient indicates improvement in QoL in patients with higher baseline values of E2

^b^A positive coefficient indicates increasing symptoms in patients with higher baseline values of E2

^c^A positive coefficient indicates decreasing symptoms in patients with higher baseline values of E2

## Discussion

The aim of the present study was to analyze the effects of letrozole on serum estradiol (E2) and estrone (E1) levels in postmenopausal breast cancer patients by using a highly sensitive and specific LC–MS/MS method. Quality of life and tolerability of the treatment were secondary outcome measures.

AIs suppress plasma estrogen levels in postmenopausal women. At menopause, mean plasma E2 levels vary from 10 to 60 pmol/L [6]. In our postmenopausal breast cancer patients, the mean E2 level measured by the LC–MS/MS method was 12 pmol/L at baseline and decreased below 5 pmol/L (LLOQ) in all patients during letrozole therapy. AI administration may lead to restoration of ovarian function in some patients with chemically induced menopause or whose ovarian function is suppressed by administration of tamoxifen as we have shown in our previous study [9]. To avoid the risk of AI failure, in the present study prior adjuvant chemotherapy was not allowed. The complete suppression of serum E2 levels reached in this study was similar to results published in the literature [7, 16].

As expected, mean E2 levels measured by routine chemiluminescent immunoassay were higher than levels obtained with LC–MS/MS. E2 measured by immunoassay at three or 12 months did not differ compared to baseline levels which is in line with our findings from a previous study [9], nor did these values show any significant association with treatment side-effects (data not shown). This indicates that E2 monitoring of AI treatment in postmenopausal women requires a highly sensitive LC–MS/MS method and direct E2 immunoassays cannot be used in reliable and accurate quantification of low E2 levels.

Baseline levels for E1 measured by a sensitive LC–MS/MS ranged from less than 5 pmol/L (LLOQ < 5 pmol/L) to 226 pmol/L with a mean of 66 pmol/L. Levels of serum E1 also decreased below the LLOQ level in 89 of 90 patients during letrozole treatment. Only in one patient, serum E1 level remained above 5 pmol/L at three months and decreased below LLOQ at 12 months.

Adjuvant AI treatment is recommended for at least five years and extended treatment for up to ten years especially for patients with high-risk features [2]. The impact of adjuvant AI treatment on QoL is part of the ongoing discussion on the treatment of breast cancer patients. AI may cause adverse events with negative impact on treatment compliance and on QoL [3]. Non-adherence in AI treatment is a well-documented problem and in this patient population it may have a negative effect on survival [17]. In our study 8% of the patients discontinued adjuvant treatment of letrozole during the first year of treatment. Overweight women discontinued letrozole treatment more often than others. Obesity is associated with increased body aromatization. At baseline, a weak statistically non-significant positive correlation between BMI and E2 levels was observed. Patients with discontinuation also had a higher baseline E2, however, the difference was not statistically significant. The majority of patients who discontinued treatment had tumors with low malignancy grade. Thus, a perceived favorable prognosis even without adjuvant treatment may have contributed to the decision to discontinue treatment.

There was no change in global health status, and all functional scales scores remained stable during the first year of adjuvant letrozole treatment. The reported overall QoL was higher than 70 points and all functional scales were higher than 80 points, which is similar to the QoL of the general population [18]. Vasomotor symptoms, body pain, joint and muscles pain, and vaginal dryness increased significantly during letrozole treatment. Similar symptoms were reported from a placebo controlled MA17 trial in which 5187 postmenopausal women who had completed 5 years of adjuvant tamoxifen therapy were randomized to a further 5 years receiving letrozole or placebo. The incidence of vasomotor symptoms and musculoskeletal symptoms was significantly higher in patients treated with letrozole compared to placebo [19]. On the other hand, the incidence of vaginal dryness was low, and not higher in the letrozole group. Treatment timing may at least partly explain the difference in vaginal side-effects, since the present trial was conducted during the first year of endocrine treatment, in contrast to the MA17 trial which recruited patients who had already been 5 years on treatment. In accordance with the present study letrozole did not have an adverse impact on overall QoL in MA17 [20].

Women with higher pre-treatment E2 levels experienced more joint and muscle pain during letrozole treatment. However, we did not find any association between pre-treatment hormone levels and the other significant side-effects. The relationship between sensitivity to pain and estrogen levels is poorly understood. However, pain sensitivity decreases during high estrogen phases of the menstrual cycle [21]. Thus, these findings suggest that estrogen deprivation may be the cause of AI-associated arthralgias.

The main strength of the current study is the use of the sensitive LC–MS/MS estrogen assay for monitoring changes in the levels of postmenopausal E2 during AI treatment. Another strength is the systematic assessment of QoL and symptoms with relevant questionnaires during AI treatment. Limitations include the relatively small number of patients, the lack of a control group, and the short one- year follow-up time.

## Conclusion

In conclusion, letrozole treatment caused complete suppression of both E2 and E1 when measured by our highly sensitive LC–MS/MS assay. A high pretreatment E2 level was associated with more frequent joint and muscle pain during letrozole treatment. Our commercial chemiluminescent immunoassay for serum E2 had no value in assessing these low estrogen levels.

## Supplementary Information

Below is the link to the electronic supplementary material.

Supplementary file1 (DOCX 14 KB)

## Data Availability

Enquiries about data availability should be directed to the authors.
