# Supplementary material for: Effects of letrozole on serum estradiol and estrone in postmenopausal breast cancer patients and tolerability of treatment: a prospective trial using a highly sensitive LC–MS/MS (liquid chromatography–tandem mass spectrometry) method for estrogen measurement
Source: Breast Cancer Res Treat. 2023 Jul 25;201(3):425–35. doi: 10.1007/s10549-023-07054-3 (PMC10460747; doi:10.1007/s10549-023-07054-3)
Supplement: Supplementary file 1 — Supplementary file1 (DOCX 14 KB) [file 10549_2023_7054_MOESM1_ESM.docx]

### Supplement 1. The effect of baseline E1 and FSH on global QoL, pain, side effects of systemic therapy, vasomotor symptoms, joint or muscle pain, and vaginal dryness during 12 months of letrozole

| **Dependent variables** | **Independent variables** | **Standardized coefficient** | **P value** |
| --- | --- | --- | --- |
| QoL (EORT QLQ-C30) ^€^ | E1 | .14 | 0.23 |
| Pain (EORT QLQ-C30) ^#^ | E1 | -.13 | 0.24 |
| Side effects of systemic therapy (EORT QLQ-BR23) ^#^ | E1 | -.07 | 0.51 |
| Vasomotor symptoms (WHQ) ^&^ | E1 | -.1 | 0.39 |
| Aching joints and muscles (KI) ^#^ | E1 | .21 | 0.06 |
| Vaginal dryness (KI) ^#^ | E1 | -.14 | 0.22 |

| **Dependent variables** | **Independent variables** | **Standardized coefficient** | **P value** |
| --- | --- | --- | --- |
| QoL (EORT QLQ-C30) ^€^ | FSH | -.05 | 0.63 |
| Pain (EORT QLQ-C30) ^#^ | FSH | -.12 | 0.28 |
| Side effects of systemic therapy (EORT QLQ-BR23) ^#^ | FSH | -.10 | 0.36 |
| Vasomotor symptoms (WHQ) ^&^ | FSH | .08 | 0.49 |
| Aching joints and muscles (KI) ^#^ | FSH | -.29 | **0.008*** |
| Vaginal dryness (KI) ^#^ | FSH | -.05 | 0.66 |

*A Bonferroni-corrected-p-value threshold of 0.01 was used in the regression analyses

### ^€^ A positive coefficient indicates improvement in QoL in patients with higher baseline values of E1 and FSH.

### ^#^ A positive coefficient indicates increasing symptoms in patients with higher baseline values of E1 and FSH

### ^&^ A positive coefficient indicates decreasing symptoms in patients with higher baseline values of E1 and FSH
